# Therapeutic effect of cardiac rehabilitation on patients with long COVID combined with coronary heart disease: a retrospective study

**DOI:** 10.3389/fcvm.2025.1560243

**Published:** 2025-07-24

**Authors:** Hui Du, Xia Du, Xinyu Lei, Gang Wang, Zhengtao Wang, Yiting Du, Fangfang Bai, Dajiang Yuan

**Affiliations:** ^1^ Department of Cardiac Rehabilitation, Shanxi Cardiovascular Hospital, Taiyuan, Shanxi, China; ^2^ Department of Cardiology, Shanxi Cardiovascular Hospital, Taiyuan, Shanxi, China; ^3^ Shanxi Cardiovascular Hospital, Taiyuan, Shanxi, China; ^4^ Department of Rehabilitation, Shanxi Bethune Hospital, Shanxi Academy of Medical Sciences, Third Hospital of Shanxi Medical University, Tongji Shanxi Hospital, Taiyuan, Shanxi, China

**Keywords:** long COVID, cardiac rehabilitation, retrospective study, sleep quality, anxiety, depression

## Abstract

**Objectives:**

To investigate the efficacy of cardiac rehabilitation in patients with long COVID combined with coronary heart disease (CHD).

**Methods:**

This retrospective study included 89 patients with long COVID, who were divided into CHD group and non-CHD group. The clinical indexes such as maximum heart rate and oxygen saturation during cardiac rehabilitation were compared between the two groups of subjects. The 6-minute walk test (6-MWT), Pittsburgh Sleep Quality Index (PSQI), 7-item Generalized Anxiety Disorder Scale (GAD-7), Nicotine Dependence Detection Scale (FTND) and the Depression Screening Scale (PHQ-9) were administered to patients. The same measures were re-administered to the patients after 3 months to explore the long-term effects of cardiac rehabilitation therapy.

**Results:**

Before receiving treatment, the maximum heart rate, maximum diastolic blood pressure and minimum SpO_2_ of the CHD group were significantly lower than those of the non-CHD group. The maximum heart rate, maximum systolic blood pressure, and 6-MWT were significantly lower before treatment than those at three months for both groups of patients. The PSQI, GAD-7, PHQ-9, and FTND scores were significantly higher before treatment than those at three months for both groups of patients. Comparison of differences between the two groups before and after treatment revealed that the CHD group had significantly worse cardiovascular measurements after SARS-CoV-2 infection than the non-CHD group.

**Conclusion:**

Patients with long COVID combined with CHD have more severe symptoms, especially for their cardiac function. Cardiac rehabilitation can effectively improve patients' symptoms, and has long-term effects.

## Introduction

Coronary heart disease (CHD) is a heart disease caused by coronary atherosclerosis that results in narrowing of the blood vessel lumen, occlusion or vasospasm, and other triggers of myocardial ischaemia and hypoxic necrosis, with a high rate of disability and death (1). In recent years, with the widespread infection of SARS-CoV-2, many patients with CHD have also become patients with long COVID (2). SARS-CoV-2 mainly invades the human respiratory system, and its clinical manifestations are primarily of the respiratory nature. However, SARS-CoV-2 also affects the cardiovascular and nervous systems (3, 4). Many patients with SARS-CoV-2 have non-specific cardiovascular symptoms (such as chest tightness, palpitations, and fatigue) remain for weeks or months. These cardiovascular symptoms are the main manifestations of long COVID (5, 6). Studies have confirmed that SARS-CoV-2 can cause myocardial injury through direct (viral invasion of cardiomyocytes) or indirect effects (viral triggering of systemic inflammatory responses, cytokine storms, and hypoxic states that damage myocardial tissue) (7–9), leading to exacerbation of cardiovascular disease. And the latest one systematic review and meta-analysis reported that the risk of cardiovascular disease increased significantly four weeks or more after recovering from acute COVID-19 (10). The latest research findings demonstrate a profound impact of SARS-CoV-2 on mitochondrial integrity, shedding light on the cardiovascular implications of Long COVID (11).

Recent literature has reported that psychosocial factors significantly influence the development and prognosis of cardiovascular disease (12). Anxiety and depression levels in the COVID-19 population are significantly higher, and patients with CHD have a worse psychological state, which may induce an acute event. Barton et al. (13) demonstrated that severe anxiety and depression can over-activate sympathetic nerves and increase cardiovascular risk. Psychological intervention in patients can help improve psychosomatic disorders and promote their recovery (14).

The mission of cardiac rehabilitation is to proactively control the risk factors for cardiovascular disease by developing individualised exercise programs and health management plans for patients with CHD, heart failure, and various cardiovascular diseases, such as coronary angioplasty or coronary artery bypass grafting. Recently, cardiac rehabilitation has been shown to reduce mortality in patients with heart failure and CHD and to reduce the risk factors for cardiovascular disease (15, 16). In many places, cardiac rehabilitation is an important treatment for patients with acute coronary syndrome (ACS), and it is also an important recommendation for patients after haemodialysis in non-ACS situations (17). Exercise-based cardiac rehabilitation is recognised as a key component of the comprehensive management of CHD and is an international guideline Class I recommendation with Level A evidence. However, there was an absence of prior studies examining the long-term effect of cardiac rehabilitation in the patients with long COVID and coronary heart disease.

In this study, we analysed retrospective data to compare the differences in clinical characteristics between patients with and without CHD during the long COVID, as well as the differences in sleep and mental status, to elaborate on the effects of long COVID on patients with CHD and to reveal the potential mechanisms by which SARS-CoV-2 infections lead to exacerbation of CHD in patients with COVID-19. Meanwhile, by comparing the follow-up data after 3 months, the long-term effect of cardiac rehabilitation was explored to provide a reference and basis for the management of cardiac rehabilitation in patients with CHD combined with long COVID.

## Methods

**Figure 1:**
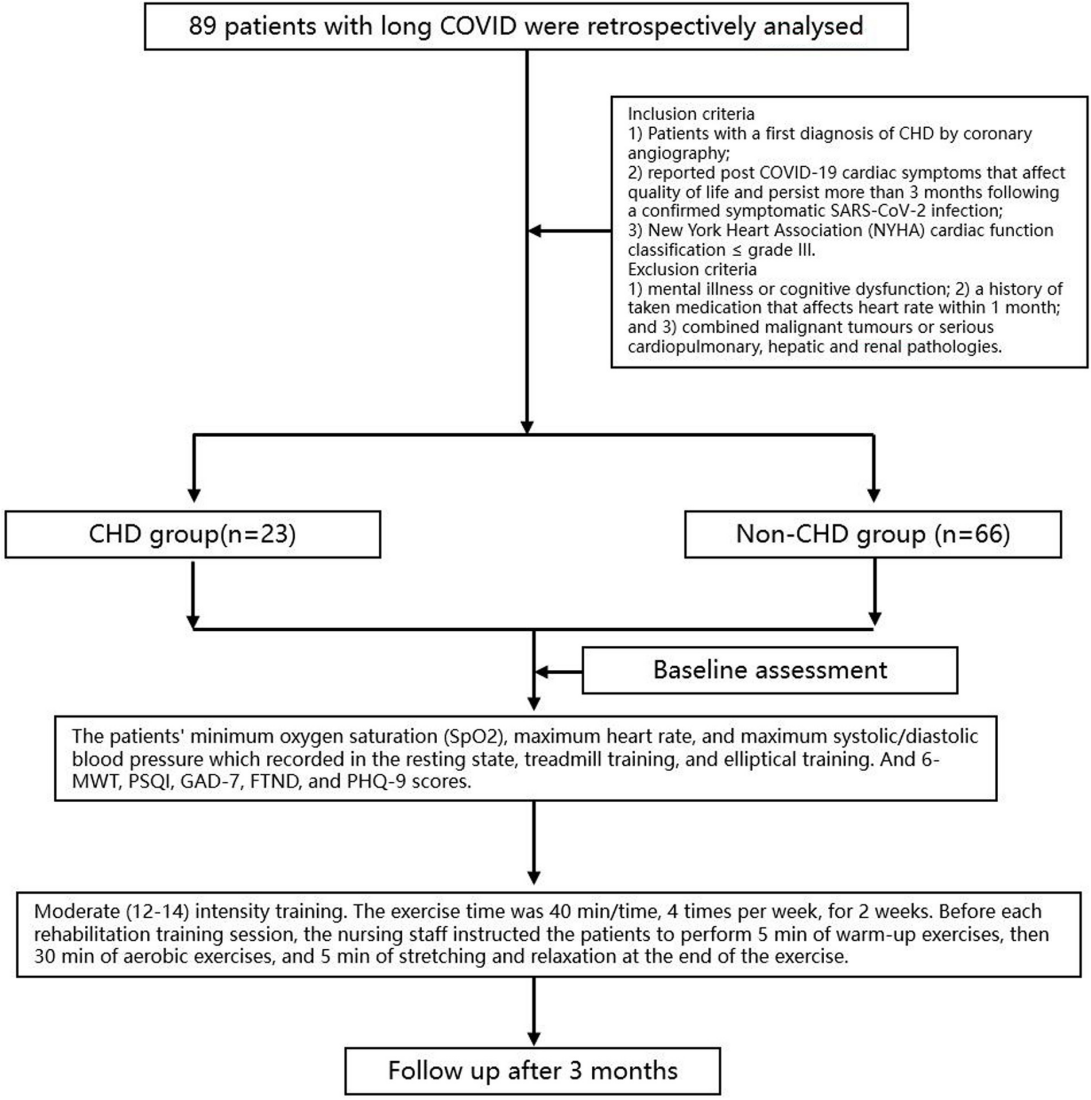
The flow diagram of study.

This is a single-center retrospective study. The detailed process was shown in Figure 1. Eighty-nine patients with long COVID who visited the Shanxi Cardiovascular Hospital between March 2023 and March 2024 were retrospectively analysed, including 23 patients with CHD in the observation group and 66 patients without CHD in the control group. This study complied with the requirements of the Declaration of Helsinki and was reviewed and approved by the Ethics Committee of the Shanxi Cardiovascular Hospital (Approval number: 2023xxg004). Inclusion criteria were patients with (1) Patients with a first diagnosis of CHD by coronary angiography; (2) reported post COVID-19 cardiac symptoms that affect quality of life and persist more than 3 months following a confirmed symptomatic SARS-CoV-2 infection; and (3) New York Heart Association (NYHA) cardiac function classification ≤grade III. Patients were excluded if they had (1) mental illness or cognitive dysfunction; (2) a history of taken medication that affects heart rate within 1 month; and (3) combined malignant tumours or serious cardiopulmonary, hepatic and renal pathologies.

### Cardiac rehabilitation

Cardiac rehabilitation during hospitalisation was based on low- and moderate-intensity aerobic exercise, and intensity was determined using the Rating of Perceived Exertion (RPE). Moderate (12–14) intensity training was adopted, and changes in the patients' vital signs were closely monitored during the treatment period. The exercise time was 40 min/time, 4 times per week, for 2 weeks. Before each rehabilitation training session, the nursing staff instructed the patients to perform 5 min of warm-up exercises, then 30 min of aerobic exercises, and 5 min of stretching and relaxation at the end of the exercise. The intensity of physical activity was limited by clinical conditions and activity tolerance, and monitored and assisted by physical therapists. Patients were also instructed and followed up after discharge from the hospital.

### Assessment indicators

General information such as age, gender, body mass index, smoking history, history of novel coronavirus infection, and past history were collected from all subjects at the time of hospital admission. Laboratory tests included red blood cell count, white blood cell count, haemoglobin, platelets, potassium, albumin, liver and kidney functions, and other clinical indexes. The patients' minimum oxygen saturation (SpO_2_), maximum heart rate, and maximum systolic/diastolic blood pressure were recorded in the resting state, treadmill training, and elliptical training.

We performed the 6-minute walking test (6-MWT), which involved measurement of the 6-minute walking distance and is a submaximal functional test indicative of performing daily activities. The safety and feasibility of this test have led to its use in cardiovascular rehabilitation. The Pittsburgh Sleep Quality Index (PSQI) is a widely used questionnaire to assess one's sleep quality for the past month, with 7 components for 7 specific features of sleep. Secondary outcomes included HDRS-17 and Self-rating Anxiety Scale scores, which provided a subjective assessment of patients' mental health state, and the actigraphy data, consisting of sleep efficiency, times of sleep awakenings, and total sleep time, which provided a relatively objective assessment of patients' sleep status. The 7-item Generalized Anxiety Disorder Scale (GAD-7) is a self-report of the severity of generalized anxiety disorder and its symptoms with 7 items. Participants rated each item on a 4-point Likert scale ranging from 0 (Not at all) to 3 (Nearly every day). A total score was calculated by summing up the item scores. The score ranges from 0 to 21 and can be categorized as 0–4 (minimal anxiety), 5–9 (mild anxiety), 10–14 (moderate anxiety), and 15–21 (severe anxiety). The Fagerström test for Nicotine Dependence (FTND)^35^ is a standard instrument for assessing the intensity of physical addiction to nicotine. It contains six items that evaluate the quantity of cigarette consumption, the compulsion to use, and dependence. In scoring the FTND for Nicotine Dependence, yes/no items are scored from 0 to 1 and multiple-choice items are scored from 0 to 3. The items are summed to yield a total score of 0–10. The higher the total Fagerström score, the more intense is the patient's physical dependence on nicotine. Depression was measured using Patient Health Questionnaire-9 (PHQ-9). The questionnaire includes 9 multiple-choice questions assessing the frequency of the actual 9 criteria upon which the diagnosis of Diagnostic and Statistical Manual of Mental Disorders, 4th ed, depressive disorders was based over the previous 2 weeks. Each question has 4 possible answers, corresponding to a score of 0–3, with higher scores indicating higher depression severity. Total scores were calculated by taking the summation of all values. A score of 0–4 indicates minimal depression; 5–9, mild depression; 10–14, moderate depression; 15–19, moderate to severe depression; and 20–27, severe depression. A change in PHQ-9 score of 5 points is considered clinically significant.

The 6-MWT, PSQI, GAD-7, FTND, and PHQ-9 were also recorded before treatment. The same assessments were repeated for all patients after 3 months to analyse the efficacy of cardiac rehabilitation therapy between the two patient groups.

### Statistical analysis

Statistical analysis was performed using SPSS 26.0 software. Measurement information was expressed as $\$\bar{x}\pm s\$$, and an independent sample *t*-test was used for comparison between groups. Counting information was expressed as rate (%), and the χ^2^ test was used for comparison between groups. A two-sided test was used, and *p* < 0.05 was considered statistically significant. Normality assumptions were checked before applying *t*-tests.

## Results

There were more male patients and more patients with a history of diabetes mellitus in the CHD group than in the non-CHD group (*p* < 0.05). There were not statistically significant differences in age, height, BMI, other previous history and laboratory indicators when comparing the subjects in the two groups (Table 1). All subjects had not reported any cardiovascular adverse events during the period of cardiac rehabilitation.

**Table 1:** Comparison of general clinical data between CHD and non-CHD groups.

Items	CHD group (*n* = 23)	non-CHD group (*n* = 66)	*t*/χ^2^	*P*
Age (years, $\$\bar{x}\pm s\$$)	62 ± 9	60 ± 8	−1.04	0.29
Sex (Male/Female)	18/5	36/30	4.02	0.04*
Height (cm, $\$\bar{x}\pm s\$$)	167.78 ± 8.31	164.77 ± 7.83	−1.56	0.12
Weight (kg, $\$\bar{x}\pm s\$$)	72.65 ± 10.03	69.55 ± 10.90	−1.58	0.11
BMI (kg/m^2^, $\$\bar{x}\pm s\$$)	26.13 ± 2.85	25.57 ± 3.16	−0.75	0.45
Antecedent history (cases)				
Hypertensive disease	11	30	0.03	0.84
Diabetes	10	14	4.29	0.03*
Hyperlipidemia	9	26	0.00	0.98
Chronic lung disease	8	14	1.68	0.19
White blood cell count (×10^9^/L, $\$\bar{x}\pm s\$$)	6.22 ± 1.57	6.34 ± 1.45	0.34	0.74
Neutrophil count (×10^9^/L, $\$\bar{x}\pm s\$$)	3.51 ± 0.94	3.86 ± 1.21	1.26	0.21
Lymphocyte count (×10^9^/L, $\$\bar{x}\pm s\$$)	1.96 ± 0.70	1.84 ± 0.59	−0.83	0.41
Monocyte count (×10^9^/L, $\$\bar{x}\pm s\$$)	0.51 ± 0.23	0.48 ± 0.19	−0.53	0.59
Hemoglobin (g/L, $\$\bar{x}\pm s\$$)	143.22 ± 14.01	140.66 ± 13.29	−0.78	0.44
Platelet count (×10^9^/L, $\$\bar{x}\pm s\$$)	206.87 ± 47.75	221.20 ± 49.85	1.20	0.23
Albumin (g/L, $\$\bar{x}\pm s\$$)	42.57 ± 4.34	42.27 ± 4.44	−0.27	0.78
Potassium (mmol/L, $\$\bar{x}\pm s\$$)	3.93 ± 0.28	3.93 ± 0.32	0.06	0.95

CHD, coronary heart disease; non-CHD, non-coronary heart disease.

*p < 0.05.

A comparative analysis of the two groups prior to receiving treatment showed statistically significant differences in maximum heart rate (*t* = −3.042, *p* = 0.002, Cohen's *d* = 0.720), diastolic blood pressure (*t* = −2.373, *p* = 0.021, Cohen's *d* = 0.560), and SpO_2_ (*t* = −2.113, *p* = 0.036, Cohen's *d* = 0.717) during walking training. During elliptical training, the maximum heart rate (*t* = −3.243, *p* = 0.002, Cohen's *d* = 0.827), maximum systolic blood pressure (*t* = −2.323, *p* = 0.023, Cohen's *d* = 0.593), diastolic blood pressure (*t* = −2.127, *p* = 0.037, Cohen's *d* = 0.543), and minimum SpO_2_ (*t* = −2.410, *p* = 0.013, Cohen's *d* = 0.360) were significantly lower in the CHD group than in the non-CHD group. Patients in the CHD group had significantly higher PSQI scores (*t* = 2.064, *p* = 0.044, Cohen's *d* = 0.207), GAD-7 scores (*t* = 2.759, *p* = 0.007, Cohen's *d* = 0.259) and PHQ-9 scores (*t* = 2.052, *p* = 0.044, Cohen's *d* = 0.373) than those in the non-CHD group (Table 2).

**Table 2:** Comparison of pre-treatment clinical data between CHD group and non-CHD group.

Items	CHD group (*n* = 23)	non-CHD group (*n* = 66)	*t*	*P*
Resting heart rate	73.93 ± 10.83	76.92 ± 8.83	−1.332	0.183
Resting systolic blood pressure	120.07 ± 15.87	124.11 ± 16.78	−1.033	0.303
Resting diastolic blood pressure	76.93 ± 10.33	76.62 ± 10.99	0.112	0.903
Resting SpO_2_	93.78 ± 1.80	94.62 ± 1.83	−1.963	0.052
Walking heart rate	101.48 ± 11.45	109.87 ± 11.75	−3.042	0.002*
Walking systolic blood pressure	148.51 ± 28.24	151.28 ± 16.63	−0.553	0.582
Walking diastolic blood pressure	83.48 ± 12.79	89.77 ± 10.35	−2.373	0.021*
Walking SpO_2_	94.81 ± 1.61	95.72 ± 1.48	−2.113	0.036*
Ellipsometer heart rate	87.09 ± 11.99	95.75 ± 9.74	−3.243	0.002*
Ellipsometer systolic blood pressure	122.36 ± 14.90	130.75 ± 13.81	−2.323	0.023*
Ellipsometer diastolic blood pressure	75.22 ± 10.25	80.71 ± 10.03	−2.127	0.037*
Ellipsometer SpO_2_	94.68 ± 1.75	95.14 ± 1.40	−2.410	0.013*
6MWT	390.81 ± 50.89	390.73 ± 46.74	0.007	0.994
PSQI	8.48 ± 1.65	7.98 ± 2.72	2.064	0.044*
GAD-7	4.84 ± 2.17	3.83 ± 3.42	2.759	0.007*
PHQ-9	5.74 ± 1.65	4.23 ± 4.73	2.052	0.044*
FTND	1.18 ± 1.88	1.00 ± 1.85	0.421	0.675

CHD, coronary heart disease; non-CHD, non-coronary heart disease; 6-MWT, 6-minute walk test; PSQI, Pittsburgh sleep quality index; GAD-7, 7-item generalized anxiety disorder scale; FTND, nicotine dependence detection scale; PHQ-9, the depression screening scale.

*p < 0.05.

Patients in the CHD group before and after treatment showed a statistically significant difference in heart rate (*t* = 3.244, *p* = 0.003, Cohen's *d* = 1.089) and SpO_2_ (*t* = −4.740, *p* = 0.001, Cohen's *d* = 0.586) measured at rest. In walking training, the differences in maximum heart rate (*t* = −3.054, *p* = 0.002, Cohen's *d* = 0.114), systolic blood pressure (*t* = −3.035, *p* = 0.003, Cohen's *d* = 0.445) and SpO_2_ (*t* = −2.133, *p* = 0.034, Cohen's *d* = 0.097) were statistically significant. In elliptical training, the maximum heart rate (*t* = −3.235, *p* = 0.002, Cohen's *d* = 0.768), systolic blood pressure (*t* = −2.443, *p* = 0.023, Cohen's *d* = 0.217) and minimum SpO_2_ (*t* = −2.160, *p* = 0.042, Cohen's *d* = 0.468) were significantly lower before treatment than after 3 months. Patients in the CHD group had significantly higher 6-MWT score after 3 months (*t* = −2.766, *p* = 0.009, Cohen's *d* = 0.764)). Likewise, patients in the CHD group had significantly higher PSQI score (*t* = 2.947, *p* = 0.007, Cohen's *d* = 0.658), GAD-7 score (*t* = 2.865, *p* = 0.008, Cohen's *d* = 0.478), PHQ-9 score (*t* = 2.656, *p* = 0.013, Cohen's *d* = 0.4435) and FTND scores (*t* = 3.124, *p* = 0.004, Cohen's *d* = 0.508) when measured 3 months after rehabilitation (Table 3).

**Table 3:** Comparison of clinical data between pre-treatment and after 3 months in CHD group.

Items	Pre-treatment of CHD (*n* = 23)	Post-treatment of CHD (*n* = 23)	*t*	*P*
Resting heart rate	73.93 ± 10.83	70.00 ± 6.20	3.244	0.003*
Resting systolic blood pressure	120.07 ± 15.87	118.26 ± 11.46	1.555	0.132
Resting diastolic blood pressure	76.93 ± 10.33	76.00 ± 8.70	1.850	0.076
Resting SpO_2_	93.78 ± 1.80	95.00 ± 0.91	−4.740	0.001*
Walking heart rate	101.48 ± 11.45	107.89 ± 8.89	−3.054	0.002*
Walking systolic blood pressure	148.51 ± 28.24	150.40 ± 23.71	−3.035	0.003*
Walking diastolic blood pressure	83.48 ± 12.79	83.51 ± 11.61	−0.042	0.991
Walking SpO_2_	94.81 ± 1.61	95.22 ± 1.15	−2.133	0.034*
Ellipsometer heart rate	87.09 ± 11.99	90.36 ± 10.29	−3.235	0.002*
Ellipsometer systolic blood pressure	122.36 ± 14.90	129.81 ± 13.43	−2.443	0.023*
Ellipsometer diastolic blood pressure	75.22 ± 10.25	75.22 ± 9.98	−0.127	0.997
Ellipsometer SpO_2_	94.68 ± 1.75	95.86 ± 1.58	−2.160	0.042*
6MWT	390.81 ± 50.89	405.33 ± 43.63	−2.766	0.009*
PSQI	8.48 ± 1.65	7.00 ± 1.03	2.947	0.007*
GAD-7	4.84 ± 2.17	2.37 ± 2.18	2.865	0.008*
PHQ-9	5.74 ± 1.65	2.33 ± 1.66	2.656	0.013*
FTND	1.18 ± 1.88	0.48 ± 0.93	3.124	0.004*

CHD, coronary heart disease; non-CHD, non-coronary heart disease; 6-MWT, 6-minute walk test; PSQI, Pittsburgh sleep quality index; GAD-7, 7-item generalized anxiety disorder scale; FTND, nicotine dependence detection scale; PHQ-9, the depression screening scale.

*p < 0.05.

Patients in the non-CHD group before and after treatment had statistically significant differences in resting heart rate (*t* = 4.792, *p* = 0.001, Cohen's *d* = 0.624), systolic blood pressure (*t* = 2.500, *p* = 0.016, Cohen's *d* = 0.299), diastolic blood pressure (*t* = 4.227, *p* = 0.001, Cohen's *d* = 0.356) and SpO_2_ (*t* = −4.269, *p* = 0.001, Cohen's *d* = 0.912). In the walking training, the difference in maximum diastolic blood pressure (*t* = 3.241, *p* = 0.002, Cohen's *d* = 0.008) was statistically significant. During elliptical training, the difference between the maximum heart rate (*t* = 5.482, *p* = 0.001, Cohen's *d* = 0.279) and SpO_2_ (*t* = −3.341, *p* = 0.002, Cohen's *d* = 0.461) was significant. The 6-MWT before treatment (*t* = −3.273, *p* = 0.003, Cohen's *d* = 0.586) was significantly lower than that at 3 months, and the PSQI score (*t* = 4.792, *p* = 0.001, Cohen's *d* = 0.567), GAD-7 score (*t* = 3.478, *p* = 0.001, Cohen's *d* = 0.551), PHQ-9 score (*t* = 3.166, *p* = 0.003, Cohen's *d* = 0.511) and FTND score (*t* = 3.700, *p* = 0.001, Cohen's *d* = 0.601) were significantly higher than that after 3 months (Table 4).

**Table 4:** Comparison of clinical data between pre-treatment and assessment after 3 months in non-CHD group.

Items	Pre-treatment of non-CHD (*n* = 66)	Post-treatment of non-CHD (*n* = 66)	*t*	*P*
Resting heart rate	76.92 ± 8.83	72.17 ± 6.25	4.792	0.001*
Resting systolic blood pressure	124.11 ± 16.78	122.09 ± 12.51	2.500	0.016*
Resting diastolic blood pressure	76.62 ± 10.99	74.66 ± 8.91	4.227	0.001*
Resting SpO_2_	94.62 ± 1.83	95.93 ± 1.30	−4.269	0.001*
Walking heart rate	109.87 ± 11.75	109.11 ± 8.88	0.830	0.410
Walking systolic blood pressure	151.28 ± 16.63	150.32 ± 14.16	1.760	0.084
Walking diastolic blood pressure	89.77 ± 10.35	88.01 ± 8.59	3.241	0.002*
Walking SpO_2_	95.72 ± 1.48	95.56 ± 11.76	0.703	0.485
Ellipsometer heart rate	95.75 ± 9.74	92.45 ± 6.79	5.482	0.001*
Ellipsometer systolic blood pressure	130.75 ± 13.81	130.39 ± 13.42	1.751	0.086
Ellipsometer diastolic blood pressure	80.71 ± 10.03	80.15 ± 8.75	1.548	0.128
Ellipsometer SpO_2_	95.14 ± 1.40	96.35 ± 1.21	−3.341	0.002*
6MWT	390.73 ± 46.74	413.73 ± 26.46	−3.273	0.003*
PSQI	7.98 ± 2.72	6.98 ± 1.58	4.792	0.001*
GAD-7	3.83 ± 3.42	3.26 ± 2.97	3.478	0.001*
PHQ-9	4.23 ± 4.73	3.39 ± 3.47	3.166	0.003*
FTND	1.00 ± 1.85	0.41 ± 0.93	3.700	0.001*

CHD, coronary heart disease; non-CHD, non-coronary heart disease; 6-MWT, 6-minute walk test; PSQI, Pittsburgh sleep quality index; GAD-7, 7-item generalized anxiety disorder scale; FTND, nicotine dependence detection scale; PHQ-9, the depression screening scale.

*p < 0.05.

A comparison of the pre- and post-treatment differences between the two groups of subjects showed that at rest, the differences in maximum heart rate (*t* = −2.215, *p* = 0.026, Cohen's *d* = 1.706) and minimum SpO_2_ (*t* = −3.279, *p* = 0.002, Cohen's *d* = 1.650) were significantly different. During walking training, the differences in maximum heart rate (*t* = −5.432, *p* = 0.001, Cohen's *d* = 0.102) and minimum SpO_2_ (*t* = −3.529, *p* = 0.001, Cohen's *d* = 0.859) were significantly different between the two groups. In elliptical training, the maximum heart rate difference (*t* = 5.482, *p* = 0.001, Cohen's *d* = 1.008), maximum systolic blood pressure difference (*t* = −5.350, *p* = 0.001, Cohen's *d* = 0.556), and minimum SpO_2_ difference (*t* = −3.254, *p* = 0.002, Cohen's *d* = 0.769) were significantly different between the two groups. The difference in 6MWT (*t* = −3.273, *p* = 0.003, Cohen's *d* = 0.762), PSQI (*t* = 4.698, *p* = 0.001, Cohen's *d* = 1.111), GAD-7 (*t* = 4.370, *p* = 0.001, Cohen's *d* = 1.033), and FTND (*t* = 3.221, *p* = 0.002, Cohen's *d* = 1.113) were significantly different (Table 5).

**Table 5:** Comparison of the post-and pre-treatment differences between the two groups.

Items	CHD group (*n* = 23)	NON-CHD group (*n* = 66)	*t*	*P*
△Resting heart rate	3.92 ± 6.28	4.75 ± 4.36	−2.215	0.026*
△Resting systolic blood pressure	1.81 ± 6.06	2.01 ± 5.87	−1.538	0.129
△Resting diastolic blood pressure	0.92 ± 2.60	1.96 ± 3.37	−1.760	0.084
△Resting SpO_2_	−1.22 ± 1.34	−0.51 ± 0.86	−3.279	0.002*
△Walking heart rate	−6.40 ± 5.91	0.75 ± 6.62	−5.432	0.001*
△Walking systolic blood pressure	−1.89 ± 11.25	0.96 ± 3.98	−0.539	0.591
△Walking diastolic blood pressure	−0.04 ± 4.57	1.75 ± 3.94	−1.744	0.085
△Walking SpO_2_	−0.41 ± 1.08	0.16 ± 0.99	−3.529	0.001*
△Ellipsometer heart rate	−3.59 ± 2.35	3.16 ± 4.25	−5.482	0.001*
△Ellipsometer systolic blood pressure	−7.45 ± 2.42	0.33 ± 1.41	−5.350	0.001*
△Ellipsometer diastolic blood pressure	0.00 ± 0.96	0.52 ± 2.48	−1.062	0.291
△Ellipsometer SpO_2_	−1.18 ± 0.32	−0.20 ± 0.45	−3.254	0.002*
△6MWT	−15.73 ± 32.44	−23.73 ± 17.36	−3.273	0.003*
△PSQI	1.48 ± 0.85	1.00 ± 1.52	4.698	0.001*
△GAD-7	1.67 ± 1.21	0.57 ± 1.18	4.370	0.001*
△PHQ-9	3.41 ± 0.80	0.83 ± 1.91	3.221	0.002*
△FTND	0.70 ± 1.17	0.58 ± 1.15	1.709	0.095

CHD, coronary heart disease; non-CHD, non-coronary heart disease; 6-MWT, 6-minute walk test; PSQI, Pittsburgh sleep quality index; GAD-7, 7-item generalized anxiety disorder scale; FTND, nicotine dependence detection scale; PHQ-9, the depression screening scale.

*p < 0.05.

## Discussion

Current studies have shown that SARS-CoV-2 infection exacerbates cardiovascular system damage, causing new heart damage or aggravating the original chronic cardiovascular disease (18, 19). In patients with prior chronic cardiovascular disease, co-infection with SARS-CoV-2 carries a worse prognosis and a higher risk of developing severe disease and death (20). In this study, we found that patients with CHD exhibited worse exercise capacity during the long COVID phase compared with patients without CHD. In particular, during moderate-intensity walking and elliptical training, patients with CHD had lower minimum oxygen saturation and a small degree of heart rate variability, suggesting reduced cardiac reserve capacity. The results of this study confirm that patients have more severe symptoms, especially with regard to cardiovascular function. These may require more time for patients to recover.

Activity tolerance is an important indicator for assessing the efficacy of rehabilitation in patients with CHD, and science-based exercise can render symptomatic improvement, while recovery of physical strength will lead to a better prognosis (21). In terms of the type of exercise, aerobic exercise is appropriate. At the same time, the duration and intensity of exercise should be gradual (22). In this study, by evaluating the changes in heart rate, blood pressure and SpO_2_ during 6-MWT, walking training and elliptical exercise, we found that the exercise capacity of the subjects in both groups increased significantly compared with that before cardiac rehabilitation training. By comparing the differences between the two groups, we found that the improvement in exercise capacity was more obvious in subjects without CHD. This result also confirms that patients with CHD have more serious cardiac function damage, a poorer prognosis, and a slower recovery process. Although there is consensus that rational exercise is beneficial for functional recovery, the current exercise guidelines for patients with CHD are still based on broad recommendations from past experience. Considering the diversity and complexity of individual patients, an accurate exercise prescription remains challenging (23).

Recent studies have shown that after infection with novel coronaviruses, the incidence of psychosomatic disorders in patients with CHD is significantly higher, mostly manifesting as psychological panic, anxiety and depression (24). In this study, by assessing the PSQI, the GAD-7, and the PHQ-9 tests of the two groups of subjects, it was found that insomnia was present in both groups to varying degrees and was accompanied by mild anxiety and depression. This result confirms that both CHD and SARS-CoV-2 infections affect the mental health and sleep of patients. These negative psychological responses cause decreased sleep quality and severe insomnia, which greatly affect the stabilisation and rehabilitation efficacy of CHD and even lead to exacerbation of the disease and adverse cardiac events. Timely and appropriate psychological care interventions can effectively reduce psychological responses to anxiety and depression in patients with CHD and improve sleep (25).

Smoking is an independent risk factor for coronary heart disease (26) and is also an independent risk factor affecting the prognosis of COVID-19. Smoking cessation reduces the risk of CHD events, and the benefits of smoking cessation are observed in the short term. Smoking cessation has become an important interventional factor in patients with cardiovascular disease; however, the current success rate of smoking cessation is unsatisfactory, and there is an urgent need for knowledge regarding the clinical characteristics of smokers with cardiovascular disease to guide healthcare professionals to develop rational tobacco control strategies. In this study, the FTND scores of the two groups of subjects revealed that both groups had a history of smoking and mild nicotine dependence. After receiving smoking cessation instructions from healthcare professionals, the nicotine dependence indices of both groups were significantly reduced, but all of participants did not quit smoking. Long-term smokers have a high degree of nicotine dependence. Therefore, when implementing smoking cessation measures, health workers should pay attention to the withdrawal symptoms of patients, use personalised coping plans to help patients cope with the discomfort that occurs during the process of quitting, and recommend visiting smoking cessation clinics for alternative treatments to reduce discomfort. Healthcare professionals should encourage patients to quit smoking when providing health education to patients with CHD, and should also strengthen the education of patients' families to create a favourable environment for patients. Patients should be encouraged to increase their initiative to seek medical attention for smoking cessation and help from healthcare professionals to increase the success rate of smoking cessation.

This study found that patients with long COVID-19 complicated with coronary heart disease suffered from severe cardiac function impairment, and severe anxiety, depression and insomnia. Cardiac rehabilitation can effectively improve patients' symptoms and has long-term therapeutic effects.

This study has some limitations. It was a single-centre study, the number of enrolled patients was small, and it is only a retrospective study, not a randomised double-blind clinical trial. Therefore, it is necessary to further expand the sample size to provide a more experimental basis for the development of a cardiac rehabilitation treatment plan for patients with long COVID combined with CHD. In the design phase of this study, our main focus was on the overall differences between CHD patients and non-CHD patients in certain specific indicators. As the sample collection was based on existing medical records, we did not match or adjust for baseline differences in patients. This imbalanced sample size and unadjusted baseline differences may have certain limitations on the interpretation of the results. Therefore, future studies can further validate our findings through methods such as matching or adjustment. We believe that despite these limitations, this study still provides valuable preliminary information for related research on coronary heart disease.

## Data Availability

The original contributions presented in the study are included in the article/Supplementary Material, further inquiries can be directed to the corresponding authors.
